# Prospective, multicentre study of external ventricular drainage-related infections in the UK and Ireland

**DOI:** 10.1136/jnnp-2017-316415

**Published:** 2017-10-25

**Authors:** Aimun A B Jamjoom, Alexis J Joannides, Michael Tin-Chung Poon, Aswin Chari, Malik Zaben, Mutwakil A H Abdulla, Joy Roach, Laurence J Glancz, Anna Solth, John Duddy, Paul M Brennan, Roger Bayston, Diederik O Bulters, Conor L Mallucci, Michael D Jenkinson, William P Gray, Jothy Kandasamy, Peter J Hutchinson, Angelos G Kolias, Aminul I Ahmed

**Affiliations:** 1 Department of Clinical Neuroscience, Western General Hospital, Edinburgh, UK; 2 Division of Neurosurgery, Department of Clinical Neurosciences, University of Cambridge and Addenbrooke’s Hospital, Cambridge, UK; 3 Department of Neurosurgery, Royal London Hospital, London, UK; 4 Department of Neurosurgery, University Hospital of Wales, Cardiff, UK; 5 Wessex Neurological Centre, University Hospitals Southampton, Southampton, UK; 6 Department of Neurosurgery, Queen’s Medical Centre, Nottingham, UK; 7 Department of Neurosurgery, Royal Victoria Infirmary, Newcastle, UK; 8 Department of Neurosurgery, Beaumont Hospital, Dublin, Ireland; 9 Division of Rheumatology, Orthopaedics and Dermatology, The University of Nottingham, Nottingham, UK; 10 Department of Neurosurgery, Alder Hey Children’s NHS Trust, Liverpool, UK; 11 Department of Neurosurgery, The Walton Centre, Liverpool, UK; 12 Institute of Translational Medicine, University of Liverpool, Liverpool, UK

## Abstract

**Objectives:**

External ventricular drain (EVD) insertion is a common neurosurgical procedure. EVD-related infection (ERI) is a major complication that can lead to morbidity and mortality. In this study, we aimed to establish a national ERI rate in the UK and Ireland and determine key factors influencing the infection risk.

**Methods:**

A prospective multicentre cohort study of EVD insertions in 21 neurosurgical units was performed over 6 months. The primary outcome measure was 30-day ERI. A Cox regression model was used for multivariate analysis to calculate HR.

**Results:**

A total of 495 EVD catheters were inserted into 452 patients with EVDs remaining in situ for 4700 days (median 8 days; IQR 4–13). Of the catheters inserted, 188 (38%) were antibiotic-impregnated, 161 (32.5%) were plain and 146 (29.5%) were silver-bearing. A total of 46 ERIs occurred giving an infection risk of 9.3%. Cox regression analysis demonstrated that factors independently associated with increased infection risk included duration of EVD placement for ≥8 days (HR=2.47 (1.12–5.45); p=0.03), regular sampling (daily sampling (HR=4.73 (1.28–17.42), p=0.02) and alternate day sampling (HR=5.28 (2.25–12.38); p<0.01). There was no association between catheter type or tunnelling distance and ERI.

**Conclusions:**

In the UK and Ireland, the ERI rate was 9.3% during the study period. The study demonstrated that EVDs left in situ for ≥8 days and those sampled more frequently were associated with a higher risk of infection. Importantly, the study showed no significant difference in ERI risk between different catheter types.

## Introduction

The insertion of an external ventricular drain (EVD) is one of the the most common neurosurgical procedures. It was first described by Claude-Nicholas Le Cat in 1744 when he punctured the ventricle and left a wick in situ for congenital hydrocephalus.^1^ Since then, the procedure has seen significant refinement in its technique, an expansion of its indications and technological advances in the materials used for drainage and insertion.^2^ In the last two decades, EVD research has focused on improving the accuracy of ventricular access and infection control. EVD-related infection (ERI) is a significant complication that can lead to increased morbidity and healthcare costs.^3^ ERI has a reported rate in the literature between 1% and 45%.^4^ This wide variation is driven by differing definitions of ERI and study methodology. A range of factors are reported to be associated with increased ERI risk including duration of drainage, cerebrospinal fluid (CSF) leak, frequency of sampling and underlying aetiology.^4–8^ Efforts to reduce ERI risk have included the introduction of EVD care bundles, the use of perioperative or continuous prophylactic antibiotics and the development of antimicrobial-impregnated catheters.^5 9–11^ Observational studies of ERI rates have been primarily retrospective and those which collected data prospectively have been restricted to single neurosurgical units (NSUs).^4 12–16^ Studies with these limitations have inherent practice and selection biases, do not accurately reflect current national practice for generating health economic models nor do they lend themselves to generating a baseline against which future multicentre randomised trials can be designed and adequately powered. Using prospectively collected data from multiple NSUs, we aimed to determine the national infection rate, assess the incorporation of current evidence into clinical practice and identify parameters associated with increased infection rates that can be interrogated in future clinical studies and be used to target preventative measures.

## Materials and methods

A prospective, multicentre observational study of EVD management and infection rates was conducted across 21 NSUs in the UK and Ireland. Primary data collection took place over 6 months between November 2014 and May 2015 followed by 30-day follow-up. The inclusion criteria were any tunnelled EVD catheter inserted in a patient of any age without evidence of pre-existing CSF infection. The study protocol was approved by the Society of British Neurological Surgeons (SBNS) Academic Committee and conducted by the British Neurosurgical Trainee Research Collaborative (BNTRC).^17^ Strengthening the Reporting of Observational Studies in Epidemiology (STROBE) checklist was used to guide the preparation of this manuscript.^18^

### Data collection and outcome measures

The BNTRC is a network of neurosurgical trainees in the UK and Ireland who conduct multicentre collaborative research with the support of the academic committee of the Society of British Neurological Surgeons.^19 20^ Within each NSU, a trainee lead coordinated local patient identification and data collection working closely with a team of collaborators, including a Consultant lead. Data were entered to the Outcome Registry Intervention and Operation Network (ORION) based at the University of Cambridge. ORION is a secure database, which complies with the Department of Health Information Governance policy and meets data security standards of the Information Governance Toolkit of the Health and Social Care Information Centre. The study protocol was approved by the audit and clinical governance committee of each participating hospital.

A range of demographic and operative parameters were captured including age, sex, underlying aetiology, primary surgeon grade, type of catheter (plain, antibiotic-impregnated or silver-bearing), length of tunnelling and frequency of subsequent CSF sampling protocol (daily, alternate days, 1–2 times per week, no sampling and unknown). The primary outcome was ERI within 30 days. A pragmatic definition of ERI was used: evidence of positive CSF culture (and/or gram stain) or clinical suspicion of ERI by managing team due to CSF pleocytosis, elevated serum inflammatory markers and clinical signs including fever, meningism and altered conscious level.^21^ Secondary outcome measures included: mortality in the NSU, functional status at discharge using the modified Rankin Score (mRS) and permanent CSF diversion at 30 days.

### Statistical analysis

The statistical approach follows the principles outlined in the study protocol.^17^ In the univariate analyses, χ² test or Fisher’s exact test was used to compare clinical variables between infected and non-infected cases and outcomes between different types of catheters. The duration of prophylactic antibiotic therapy and time to infection was compared using the Kruskal-Wallis test. In the multivariate analyses of ERIs, prespecified independent variables were entered which included type of catheter, length of tunnelling, CSF sampling frequency and dichotomised duration of EVD use around the median. These were chosen because of their known or hypothesised association with ERI. To examine the rate of ERIs, HRs for ERI were calculated using Cox regression model adjusted for age, sex, type of catheter, length of tunnelling, CSF sampling frequency and dichotomised duration of EVD use. The time-at-risk period was from date of EVD insertion to ERI, death or 30 days after EVD insertion. To examine factors associated with ERIs a multiple logistic regression model using the prespecified independent variables was used. As a sensitivity analysis to test whether NSU influenced the odds of infection within 30 days of EVD insertion, a multilevel mixed effect logistic regression was performed using hospitals as clusters. This model was compared with a single-level multiple logistic regression model using the likelihood ratio test. We used STATA V.13.0 (StataCorp) for conducting statistical tests and generating graphical outputs. A p value of <0.05 denoted statistical significance.

## Results

### Demographic and operative data

During the 6-month period, a total of 495 EVD catheters were inserted in 452 patients. The follow-up was 12 308 days (median 30 days; IQR 23–30). The median age at time of insertion was 54 years and 261 (52.7%) catheters were inserted in female patients. Hydrocephalus secondary to a neurovascular aetiology (64.9%) was the the most common indication for EVD insertion followed by tumours (17.8%) and trauma (7.1%). The baseline demographic characteristics of the cohort are presented in table 1.

**Table 1:** Demographic data for 495 EVD catheters

Patient characteristics	No.	%
Age		
Mean (SD)	51.8 (19.2)	–
Median (IQR)	54 (41–67)	–
Age groups		
0–17	37	7.4
18–29	34	6.8
30–39	45	9.1
40–49	78	15.8
50–59	113	22.8
60–69	103	20.8
70–79	66	13.3
80–89	15	3.0
90+	4	1.0
Gender		
Female	261	52.7
Male	234	47.3
Pathology		
Congenital, IIH and NPH	12	2.4
Shunt dysfunction	19	3.8
Neurovascular	321	64.9
Other	20	4.0
Trauma	35	7.1
Tumour	88	17.8
Preoperative ASA status		
1	117	23.6
2	108	21.8
3	111	22.4
4	131	26.5
5	28	5.7

ASA, American Society of Anaesthesiologists; EVD, external ventricular drain; IIH, idiopathic intracranial hypertension; NPH, normal pressure hydrocephalus.

The majority (98.6%) of EVDs were inserted in the operating theatre and the remainder (1.4%) were inserted in the intensive care unit. In most cases (85.3%) prophylactic systemic antibiotics were given at induction. A small number (3.2%) had prolonged systemic antibiotic prophylaxis with the remaining groups either already on antibiotics (3.8%) or receiving no systemic antimicrobial prophylaxis (7.7%). Neurosurgical trainees were the primary surgeon in 424 (85.7%) EVD procedures with 66% of the total being inserted by senior trainees. Of the catheters inserted, 188 (38%) were antibiotic-impregnated, 161 (32.5%) were plain and 146 (29.5%) were silver-bearing catheters. EVDs remained in situ for a total of 4700 days (median of 8 days (IQR 4–13)). Table 2 presents operative data regarding the EVD procedures.

**Table 2:** Operative data for 495 EVD catheters

Operative characteristics	No.	%
Length of tunnelling		
0–5 cm	119	24.0
5–10 cm	359	72.5
>10 cm	17	3.4
Postoperative CT head scan		
No	100	20.2
Yes	395	79.8
CSF sampling frequency		
No sampling	129	26.1
1–2 times per week	234	47.3
Alternate days	24	4.9
Daily	7	1.4
Unknown	101	20.4
Catheter type		
Plain	161	32.5
Antibiotic-impregnated	188	38
Silver-bearing	146	29.5
Primary surgeon		
Intern/Foundation doctor	5	1.0
Junior neurosurgery trainee	97	19.6
Senior neurosurgery trainee	327	66.1
Consultant/Attending	66	13.3

CSF, cerebrospinal fluid; EVD, external ventricular drain.

### Infection outcome

#### Infection rate

Forty-six ERIs were observed during the follow-up period. The overall risk of infection was 9.3% (n=46/495) within 30 days. One ERI had a missing date of infection. Of the remaining 45 ERIs, six occurred after EVD removal. Therefore, the rate of ERI while an EVD was in place was 0.8% per day. The median time to infection was 9 days (IQR 5–15). For 25 cases of ERI, pathogens were isolated with a total of 29 organisms. The most common organisms were coagulase negative staphylococcus (n=10, 34.5%), followed by *Staphylococcus aureus* (n=6, 20.7%) and *Enterococcus* spp. (n=3, 10.3%). Table 3 includes the frequency of all isolated microbiological organisms.

**Table 3:** List of organisms cultured from CSF samples

Cultured organisms	No.	%
Coagulase-negative staphylococcus*	10	34.5
*Staphylococcus aureus*	6	20.7
*Enterococcus* spp.	3	10.3
*Enterobacter* spp.	2	6.9
*Klebsiella pneumoniae*	2	6.9
*Morganella morganii*	1	3.4
*Escherichia coli*	1	3.4
*Klebsiella* sp.	1	3.4
*Pseudomonas aeruginosa*	1	3.4
*Corynebacterium* sp.	1	3.4
*Streptococcus* sp.	1	3.4

*Three cases due to *Staphylococcus epidermidis*.

CSF, cerebrospinal fluid.

The median length of treatment with antibiotics was 10 days (IQR 7–14). A comparison of infected versus uninfected cases using the Χ^2^ test demonstrated that a significantly higher percentage of infected cases had a catheter in situ for ≥8 days (77.8%) compared with uninfected cases (47.3%) (p<0.01). Similarly, a significantly higher percentage of infected cases had their EVD sampled more frequently (daily or on alternate days) compared with uninfected cases (p<0.01). Conversely, there was no significant difference between infected and uninfected cases with regard to the different catheter types (p=0.31), length of tunnelling (p=0.71) or the likelihood of permanent CSF diversion at 30 days (p=0.91). Table 4 provides comparison between the infected and uninfected cases.

**Table 4:** Comparison between infected and uninfected catheters

	Infection within 30 days		p Value
	No (%)	Yes (%)	
Catheter type			
Plain	149 (92.5)	12 (7.5)	0.314
Antibiotic-impregnated	174 (92.6)	14 (7.4)	
Silver-bearing	126 (86.3)	20 (13.7)	
CSF sampling frequency			
No sampling	129 (100)	0 (0)	<0.01
1–2 times per week	211 (90.2)	23 (9.8)	
Alternate days	15 (62.5)	9 (26.5)	
Daily	3 (42.9)	4 (57.1)	
Unknown	91 (90.1)	10 (9.9)	
Length of tunnelling			
0–5 cm	106 (89.1)	13 (10.9)	0.71
5–10 cm	328 (91.4)	31 (8.6)	
>10 cm	15 (88.2)	2 (11.8)	
Primary surgeon			
Intern/Foundation doctor	4 (80.0)	1 (20.0)	0.25
Junior neurosurgery trainee	87 (89.7)	10 (10.3)	
Senior neurosurgery trainee	294 (89.9)	33 (10.1)	
Consultant	64 (97.0)	2 (3.0)	
Duration of EVD use*			
0–7 days	236 (95.9)	10 (4.1)	<0.01
8+days	212 (85.8)	35 (14.2)	
CSF diversion			
Permanent CSF diversion	71 (91.0)	7 (9.0)	0.91
No permanent diversion	378 (90.6)	39 (9.4)	

Comparisons made using Χ^2^ test.

*Two cases excluded as no valid exit date.

CSF, cerebrospinal fluid; EVD, external ventricular drain.

#### Comparison between EVD catheter types infection outcome

A Χ^2^ test for homogeneity showed there was no significant difference between the distribution of catheter types across the cohort age groups, gender and underlying pathology. A comparison of the infection rate according to catheter types showed that silver-bearing catheters had the highest infection rate (13.7%), compared with plain (7.5%) and antibiotic-impregnated catheters (7.4%), but this did not reach statistical significance (p=0.09). The median time to infection was longest for the antibiotic-impregnated catheters (11 days) followed by plain catheters (8 days) and then silver-bearing (7 days). Infections on a background of antibiotic-impregnated catheters received antibiotic treatment for a median of 14 days, silver-bearing catheters received a median of 10 days’ therapy and plain catheters received 7.5 days. Table 5 provides a summary of clinical and infection variation between the catheter subtypes.

**Table 5:** Comparison of clinical and infection parameters between catheter subtypes

	Plain (n=161)		Antibiotic-impregnated (n=188)		Silver-bearing (n=146)		p Value
	No.	%	No.	%	No.	%	
30-day infection							
No	149	92.6	174	92.6	126	86.3	0.09
Yes	12	7.5	14	7.4	20	13.7	
Time to infection (median)							
	8 (1–29)	–	11 (2–29)	–	7 (1–39)	–	0.19
Length of antibiotic therapy for ERI (median)							
	7.5 (1–28)	–	14 (1–87)	–	10 (1–37)	–	0.23
Cultured microorganisms							
Coagulase-negative staphylococcus (p)	4	36.3	2	25.0	4	40.0	–
*Staphylococcus aureus* (p)	3	27.3	2	25.0	1	10.0	–
*Enterococcus* spp. (p)	1	9.1	2	25.0	–	–	–
*Enterobacter* spp. (n)	–	–	1	12.5	1	10.0	–
*Klebsiella pneumoniae* (n)	1	9.1	–	–	1	10.0	–
*Morganella morganii* (n)	–	–	–	–	1	10.0	–
*Escherichia coli* (n)	1	9.1	–	–	–	–	–
*Klebsiella* (n)	1	9.1	–	–	–	–	–
*Pseudomonas aeruginosa* (n)	–	–	–	–	1	10.0	–
*Corynebacterium* (p)	–	–	1	12.5	–	–	–
*Streptococcus* spp. (p)	–	–	–	–	1	10.0	–
30-day mortality*							
No	100	70.4	145	81.0	93	71.0	0.05
Yes	42	29.6	34	19.0	38	29.0	
30-day mRS*							
Good (mRS 0–2)	50	35.2	79	44.1	35	26.7	0.01
Poor (mRS 3–5)	50	35.2	66	36.9	58	44.3	
Death	42	29.6	34	19.0	38	29.0	

*30-day mortality and 30-day mRS based on 452 non-duplicate patients; (p)=gram stain positive; (n)=gram stain negative.

ERI, external ventricular drain-related infection; mRS, modified Rankin Score.

#### Clinical variables associated with infection

**Figure 1:**
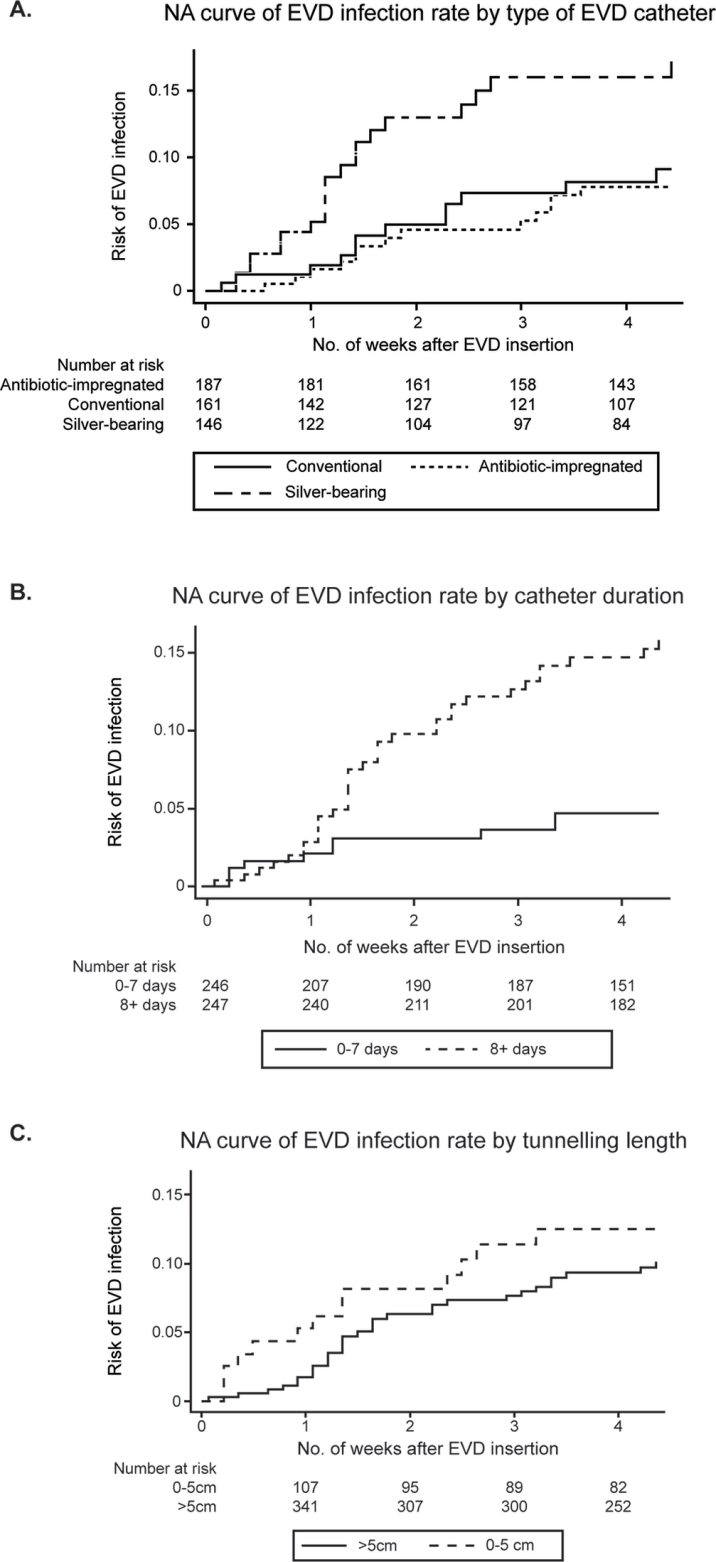
Nelson-Aalen cumulative hazard estimate of EVD infection by (A) type of EVD catheter: log-rank test of equality showed p=0.06, (B) catheter duration: log-rank test of equality showed p<0.01, (C) tunnelling length: log-rank test of equality showed p=0.41. EVD, external ventricular drain.

There was a significant increase in risk of infection for both daily and alternate day sampling regimens compared with catheters that were sampled 1–2 times per week. Similarly, catheters left in situ for>8 days had a higher risk of ERI compared with those removed at 7 days or less (OR=2.54 (95% CI 1.14 to 5.7); p=0.02). There was no association between the underlying pathology and the risk of infection in univariate analysis (p=0.76). Catheters tunnelled>5 cm had an ERI OR of 0.75 ((95% CI 0.3 to 1.45); p=0.29) compared with those with shorter tunnelling. Nelson-Aalen cumulative hazard estimate curves of catheter type, EVD duration and length of tunnelling are shown in figure 1. To determine whether NSU had an effect on ERI, a likelihood ratio test, comparing a multilevel mixed effects logistic regression using hospitals as clusters, was performed; ERI rate did not significantly vary between hospitals (p=0.16). A Cox regression model adjusted for age, sex, type of catheter, length of tunnelling, CSF sampling frequency and duration of EVD use based on 441 EVDs where time-at-risk was available identified a higher rate of infection for catheters kept in situ for 8 days or longer (HR=2.47 (95% CI 1.12 to 5.45); p=0.03). Using a similar analysis, there was no significant difference in ERI risk between catheter types (table 6).

**Table 6:** HRs for EVD infection rate using Cox regression model

	HR	95% CI	p Value
Catheter type			
Plain	Ref	–	–
Antibiotic-impregnated	0.87	0.37 to 2.03	0.75
Silver-bearing	1.35	0.63 to 2.88	0.44
Length of tunnelling			
0–5 cm	Ref	–	–
>5 cm	0.75	0.36 to 1.55	0.44
CSF sampling frequency			
1–2 times per week	Ref	-	-
Alternate days	5.28	2.25 to 12.38	<0.01
Daily	4.73	1.28 to 17.42	0.02
Unknown	1.26	0.56 to 2.82	0.58
Duration of EVD use			
0–7 days	Ref	-	-
≥8 days	2.47	1.12 to 5.45	0.03

*Cox regression model adjusted for age and sex based on 441 EVDs where date of exit (infection, death or 30 days) was available.

CSF, cerebrospinal fluid; EVD, external ventricular drain.

### Paediatric subanalysis

Among the 37 catheters inserted into patients aged<18, there were three infections giving the paediatric cohort an infection rate of 8.1%. The distribution of pathologies between adult and paediatric cases is shown in table 7. There was a significant (p<0.01) difference in the pathological aetiology necessitating an EVD insertion between the different age groups. Similarly, there was a significant difference of the catheter types inserted into the two cohorts. Particularly, antibiotic-impregnated catheters were preferred in the paediatric cohort constituting 62.2% of catheters compared with only 36% of catheters in adults. Importantly, exclusion of the paediatric subcohort did not cause a change in the observed association trends in the Cox regression analysis. Catheter drainage>8 days and sampling frequency (daily and alternate days) remained significantly associated with increased ERI risk.

**Table 7:** Comparison between paediatric and adult cohort pathology and EVD type

	Paediatrics	Adults	p Value
Pathology			<0.01
Congenital	4 (10.8)	8 (1.8)	
Shunt dysfunction	3 (8.1)	16 (3.5)	
Neurovascular	8 (21.6)	313 (68.4)	
Other	3 (8.1)	17 (3.7)	
Trauma	5 (13.5)	30 (6.6)	
Tumour	14 (37.8)	74 (16.2)	
Catheter types			<0.01
Conventional	5 (13.5)	156 (34.1)	
Antibiotic-impregnated	23 (62.2)	165 (36.0)	
Silver-bearing	9 (24.3)	137 (29.9)	

EVD, external ventricular drain.

### Mortality and functional outcome

There were 114 (25.2%) deaths observed within 30 days after EVD insertion in the 452 patients recruited in our study (table 5). Of the surviving patients, 164 (48.5%) had a good functional outcome (mRS 0–2) and 174 (51.5%) had a poor outcome (mRS 3–4).

## Discussion

Using a network of neurosurgical trainees and the BNTRC infrastructure, we were able to capture data on a national level and provide the largest prospective multicentre observational study of EVD management and infection rate in the literature. We confirmed 46 ERIs from 495 catheters giving an infection risk of 9.3% (or 9.8 ERIs per 1000 catheter days). This falls within the wide range of quoted infection risk within the literature; however, direct comparison is limited due to the differing definitions of ERIs used. We chose a pragmatic definition which included culture-positive CSF or other features of ERI (CSF pleocytosis, raised inflammatory markers, clinical features of meningitis) that prompted treatment. This definition is used in a range of studies including a previously published randomised trial assessing the effectiveness of silver-bearing catheters.^21^ A meta-analysis by Ramanan and colleagues looked at ERIs and found a total of 35 observational studies reported an overall infection rate of 11.4 ERIs per 1000 catheter days.^4^ Within their study, the authors found several definitions of infection rates used by the 35 studies and the pragmatic definition used in our study was the second most used definition (seven studies). The meta-analysis of these seven studies found a total of 17 ERIs per 1000 catheter days (95% CI 10 to 24.1). This value was double our findings of 9.8 ERIs per 1000 catheter days. This comparison, despite its limitations, reflects that the UK and Ireland’s ERI rate is consistent with the literature and may be lower than studies with an equivalent ERI definition.

One of our key findings was an increased ERI rate with a longer duration of EVD placement. Catheters in situ for≥8 days were associated with a greater than twofold increase in rate of developing an ERI compared with those in place for 7 days or less. The literature has conflicting evidence on the relationship between the length of EVD placement and ERI. Some studies have shown no association with duration of EVD placement and ERI,^22^ while others corroborated our findings.^7 8^ Moreover, some authors have recommended routine replacement of EVDs,^23^ while others argue against this.^6^ Wong and colleagues interrogated this question in a small RCT where they compared routine EVD change (every 5 days) to no change.^24^ The study found no difference in ERIs but a trend to higher infection in the routine change group; the authors advised against the use of regular pre-emptive EVD replacement. Our data also showed that an increased frequency of CSF sampling was found to be a significant parameter associated with ERI risk. In keeping with this, several studies have found that patients with an ERI were sampled more frequently than those without an ERI.^6 7^ Their statistical approaches may have been biased by the fact that the number of CSF samples increased with the duration of EVD placement. Our approach did not use absolute numbers of samples taken but rather the sampling frequency protocol. Though this approach has its benefits, we are unable to conclude a direct causal relationship between sampling frequency and ERI. When assessed in the Cox regression model, we found that both catheter duration and sampling frequency were associated with a significantly higher ERI risk. Based on the literature and our data, we conclude that catheters should be removed as early as possible and a lower frequency sampling protocol should be considered as this may help reduce ERI risk.

The development of antimicrobial impregnated catheters (both antibiotic-bearing and silver-bearing catheters) has been a major approach for combating ERI risk. The most commonly used antibiotic-impregnated catheters contain clindamycin and rifampicin, while the silver-bearing catheters contain silver nanoparticles. These catheters impede and kill bacteria with the goal of reducing ERI risk and several studies have attempted to assess their effectiveness. Despite heterogeneity, meta-analyses of these data have broadly pointed towards the effectiveness of impregnated catheters compared with plain catheters.^9 10^ Interestingly, our study showed that despite this body of evidence, almost a third of catheters inserted in the UK and Ireland were plain catheters. Our study also failed to demonstrate a significant difference in infection risk between the three different catheter types after adjusting for a range of clinical variables, although there was a trend towards silver-bearing catheters having the highest ERI rate. This is in contrast to a previous trial that suggest silver-bearing catheters are more effective than plain.^21^ In meta-analyses, antibiotic-impregnated catheters have shown more pronounced reductions in ERI risk compared with plain catheters when compared with reductions of risk seen in silver versus plain catheters.^9 10^ This reflects evidence from central venous catheters that found superiority of antibiotic-impregnated catheters compared with those coated with silver in reducing bloodstream infections.^25^ In the UK and Ireland, a major multicentre RCT (The British Antibiotic and Silver Impregnated Catheters for ventriculoperitoneal Shunts trial; BASICS trial) is currently under way aiming to assess the comparative efficacy of antibiotic-bearing, silver-bearing and plain catheters in reducing CSF infections for patients with a ventriculoperitoneal shunt.^26^ The results of this trial will be of great interest to the field and help guide future trial designs looking at a similar question in EVD care. While our data do not support the routine use of antibiotic-bearing and silver-bearing EVD catheters to reduce ERI risk, it highlights an important cost implication that warrants further study since antibiotic-bearing and silver-bearing catheters are substantially more expensive.

In our study, the majority of cultured organisms were gram-positive (68.9%) and staphylococci were the most common agent (55.1%). The predominance of staphylococci mirrors other findings in the literature.^7 8 27^ Most cultured organisms from all three catheter types were gram-positive organisms: plain catheters (63.6%), antibiotic-impregnated catheters (87.5%) and silver-bearing catheters (60%). These findings run counter to trends described in the literature of a shift towards gram-negative organisms.^6 12 28^ A range of explanations has been proposed including the impact of antimicrobial-impregnated catheters. In vitro investigation of silver-bearing catheters found a greater eradication of **Staphylococcus* epidermidis* compared with **Escherichia* coli*.^29^ It is of interest that in our data the two cases of Enterobacter were in patients with antibiotic-impregnated and silver-bearing catheters. Atkinson and colleagues concluded that their observed increase in gram-negative organisms was related to the use of silver-bearing catheters; however, their small sample placed limitations on this conclusion.^6^ Prolonged systemic prophylactic antibiotics during drainage has also been implicated in a shift towards more gram-negative microorganisms.^28 30^ In our study, only a small percentage (3.2%) of cases received prolonged systemic antibiotics which may, in part, explain the dominance of gram-positive organisms.

This study has a number of limitations. First, we were unable to confirm case ascertainment in individual NSUs. In part, this was due to the nature of rotational neurosurgical training which meant trainees would move units, not allowing for continuous data collection in some units. This may have led to an under-reporting of inserted EVDs which could have led to bias in the results. Second, our choice of ERI definition could be open to criticism as it places an onus on the managing clinician to determine the presence of ERIs in cases without a positive CSF culture. This may overestimate the number of ERIs in our cohort due to the presence of false positives. However, as argued above, we believe that the definition is pragmatic, holds relevance for day-to-day clinical practice and has been used in previous RCTs.^21^ Third, NSUs may have different protocols for EVD management that influence the risk of ERIs. In particular, a strict infection control protocol for manipulation of EVD is shown to be associated with a lower risk of ERIs.^31 32^ Local EVD management protocols may confound the results shown in our study. This confounding effect is not likely to have had a large impact on our results since our sensitivity analysis did not show a significant difference in local infection rates, but this aspect of EVD care should be taken into account when interpreting the results. Coupled to this, we did not capture data on concomitant infections which may act as a confounder. Within the data, we also found a significant difference in mortality between catheter subtypes. Though an interesting finding, our study was not designed to examine this question and caution should be taken in drawing conclusions around this observation which is at risk of confounders. Finally, many NSUs favour a single catheter type for EVD insertion. Therefore, factors specific to individual units which contribute to infection rate may confound the overall infection risk difference between the three catheter subtypes. While we did not demonstrate a significant difference between the catheter subtypes, the ERIs between the three groups allow us to perform adequate power calculations for future randomised trials.

## Conclusion

This study demonstrated an ERI rate of 9.3% in 21 centres in the UK and Ireland with a predominance of gram-positive organisms. Patients with an EVD left in situ for ≥8 days and who underwent more frequent sampling had a higher risk of infection. Importantly, there was wide variation in the choice of catheter across the country with no significant difference in ERI risk between the types. These findings support the need for a prospective clinical trial to assess the comparative effectiveness of EVD catheter type on ERI risk.
